# Supplementary material for: Virus-derived peptide inhibitors of the herpes simplex virus type 1 nuclear egress complex
Source: Sci Rep. 2021 Feb 18;11:4206. doi: 10.1038/s41598-021-83402-x (PMC7893173; doi:10.1038/s41598-021-83402-x)
Supplement: Supplementary file 1 — Supplementary Information. [file 41598_2021_83402_MOESM1_ESM.docx]

**Supporting Information**

**Virus-derived peptide inhibitors of the herpes simplex virus type 1 nuclear egress complex**

Elizabeth B. Draganova^1^ and Ekaterina E. Heldwein^1,*^

^1^Department of Molecular Biology and Microbiology, Tufts University School of Medicine, Boston, MA, 02111, USA


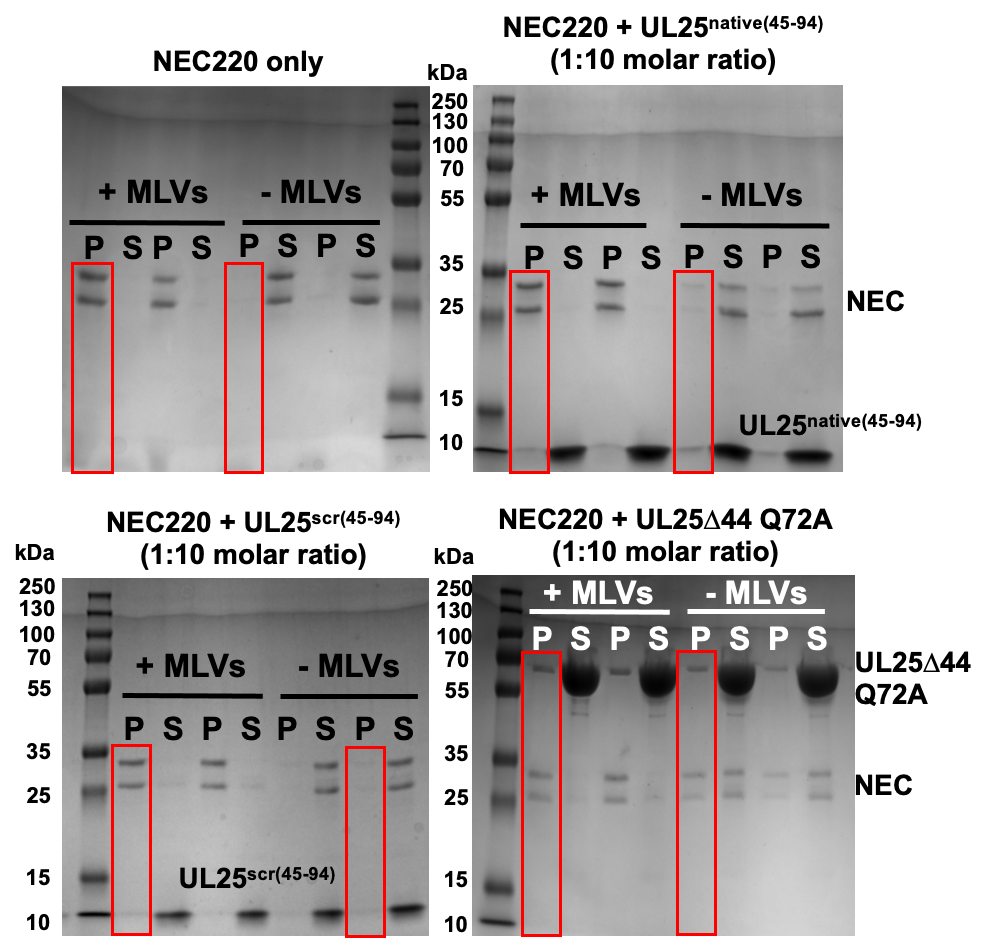


**Supplementary Fig. S1. Original SDS-PAGE images of the co-sedimentation assay samples shown in Figure 5.** Each gel represents one biological replicate used to generate the bar graph in Figure 5. All samples were run in parallel. Red boxes denote the gel lanes shown in the main figure. Gels were stained with Coomassie G-250. Gels were imaged together using a Syngene G:BOX. Both the pellet (P) and supernatant (S) samples, either in the presence (+MLVs) or absence (-MLVs) of MLVs are shown on the gel.

**Supplementary Fig. S2. Helical wheel projections of the (EAEKAAK)_7_EAEK, UL25^native(45-94)^ and UL25^scr(45-94)^ peptides. a)** The (EAEKAAK)_7_EAEK peptide is amphipathic as shown by the distribution of charged (blue and red) residues on one face of the helix versus the hydrophobic alanine residues (grey) on the other. **b)** The UL25^native(45-94)^ and UL25^scr(45-94)^ peptides are less amphipathic as indicated by the less even distribution of charged (blue and red) and polar residues (pink, light blue, and purple) versus hydrophobic (grey and yellow) residues. The N and C on each wheel indicate the N and the C termini. Wheel projections were made using Heliquest^1^.

**Supplementary Table S1. Secondary structure content (%) estimation of peptides**. Spectral deconvolution using DichroWeb^2^ was performed on samples in the absence or presence of 30% trifluoroethanol (TFE). Spectra were deconvoluted using the CONTINLL analysis program^3^. Data from reference datasets 4, 7 and SMP180 were averaged to obtain secondary structure estimates^4^.

| **Peptide** | **No TFE** | | | **30% TFE** | | |
| --- | --- | --- | --- | --- | --- | --- |
|  | **α-Helix** | **β-sheet** | **Random coil** | **α-Helix** | **β-sheet** | **Random coil** |
|  | **%** | | | | | |
| **UL25^native(45-94)^** | 12 | 12 | 76 | 37 | 12 | 51 |
| **UL25^scr(45-94)^** | 8 | 26 | 66 | 31 | 16 | 53 |
| **UL25^74-94^** | 7 | 35 | 58 | 7 | 35 | 58 |
| **gB^103-152^** | 8 | 26 | 66 | 13 | 24 | 63 |
| **(EAEKAAK)_7_EAEK** | 63 | 3 | 29 | 75 | 2 | 23 |

**Supplementary Table S2.** **List of primers and gBlock sequence used for cloning procedures described in Materials and Methods.** All primers are listed in the 5’-3’ direction. Restriction sites are underlined and mutations are bolded.

| **Primer Name** | **Primer Sequence (5’-3’)** | **Restriction Site** |
| --- | --- | --- |
| UL25 rev (JB134) | aaaaaactcgagttattacactgcgctcagatactgagg | XhoI |
| UL25Δ44 Q72A (ED06) | aatgcagcaatg**gcg**gcagcc | Site-directed |
| UL25Δ44 Q72A (ED07) | ggctgc**cgc**cattgctgcatt | Site-directed |
| (EAEKAAK)_7_EAEK peptide gBlock sequence | aaaaaa ggatcc gaa gcg gaa aaa gcg gcg aaa gaa gcg gaa aaa gcg gcg aaa gaa gcg gaa aaa gaa gcg gaa aaa gcg gcg aaa gaa gcg gaa aaa gcg gcg aaa gaa gcg gaa aaa gaa gcg gaa aaa gcg gcg aaa gaa gcg gaa aaa gcg gcg aaa gaa gcg gaa aaa taa taa ctcgag aaaaaa | BamHI/XhoI |

**Supplementary Table 3. Raw data and background values collected for each reported GUV budding assay.** The reported biological replicate average values (%) are the points presented in Figure 2.

| **Biological Replicate** | **Technical Replicate** | **Background**  **(BG)** | **ILVs/GUVs** | **Raw Value**  **(ILV-BG)** | **Average (AVG)** | **ILVs/GUVs** | **Raw Value**  **(ILV-BG)** | **Average (AVG)** | **% Normalized Budding**  **(Raw values/NEC AVG) * 100** | **Biological Replicate Average**  **(%)** |
| --- | --- | --- | --- | --- | --- | --- | --- | --- | --- | --- |
|  |  |  | **NEC220** | | | **NEC220 + UL25^native(45-94)^**  **(1:0.1 NEC:peptide)** | | |  |  |
| 1 | 1 | 10/100 = 0.10 | 17/100 = 0.17 | 0.07 | 0.09 | 13/100 = 0.13 | 0.03 | 0.10 | 34 | 112 |
|  | 2 | 6/100 = 0.06 | 15/100 = 0.15 | 0.09 |  | 19/95 = 0.20 | 0.14 |  | 161 |  |
|  | 3 | 8/100 = 0.08 | 18/100 = 0.18 | 0.10 |  | 19/95 = 0.20 | 0.12 |  | 138 |  |
| 2 | 1 | 3/96 = 0.03 | 23/99 = 0.23 | 0.20 | 0.15 | 20/99 = 0.20 | 0.17 | 0.17 | 112 | 111 |
|  | 2 | 7/95 = 0.07 | 22/100 = 0.22 | 0.15 |  | 26/100 = 0.26 | 0.19 |  | 122 |  |
|  | 3 | 9/100 = 0.09 | 20/100 = 0.20 | 0.11 |  | 24/100 =0.24 | 0.15 |  | 98 |  |
| 3 | 1 | 6/100 = 0.06 | 19/100 = 0.19 | 0.13 | 0.14 | 19/100 = 0.19 | 0.13 | 0.13 | 95 | 95 |
|  | 2 | 7/100 = 0.07 | 19/100 = 0.19 | 0.12 |  | 19/90 = 0.21 | 0.14 |  | 103 |  |
|  | 3 | 2/100 = 0.02 | 18/100 = 0.18 | 0.16 |  | 14/100 = 0.14 | 0.12 |  | 88 |  |
|  |  |  | **NEC220** | | | **NEC220 + UL25^native(45-94)^**  **(1:0.3 NEC:peptide)** | | |  |  |
| 1 | 1 | 3/96 = 0.03 | 23/99 = 0.23 | 0.20 | 0.15 | 15/100 = 0.15 | 0.12 | 0.11 | 78 | 71 |
|  | 2 | 7/95 = 0.07 | 22/100 = 0.22 | 0.15 |  | 18/100 = 0.18 | 0.11 |  | 70 |  |
|  | 3 | 9/100 = 0.09 | 20/100 = 0.20 | 0.11 |  | 19/100 = 0.19 | 0.10 |  | 67 |  |
| 2 | 1 | 6/100 = 0.06 | 19/100 = 0.19 | 0.13 | 0.14 | 14/100 = 0.14 | 0.08 | 0.9 | 59 | 68 |
|  | 2 | 7/100 = 0.07 | 19/100 = 0.19 | 0.12 |  | 13/100 = 0.13 | 0.06 |  | 44 |  |
|  | 3 | 2/100 = 0.02 | 18/100 = 0.18 | 0.16 |  | 16/100 = 0.16 | 0.14 |  | 102 |  |
| 3 | 1 | 1/100 = 0.01 | 17/90 = 0.19 | 0.18 | 0.14 | 14/100 = 0.14 | 0.13 | 0.11 | 91 | 75 |
|  | 2 | 6/100 = 0.06 | 18/100 = 0.18 | 0.14 |  | 14/100 = 0.14 | 0.08 |  | 56 |  |
|  | 3 | 5/100 = 0.05 | 18/100 = 0.18 | 0.13 |  | 16/100 = 0.16 | 0.11 |  | 77 |  |
|  |  |  | **NEC220** | | | **NEC220 + UL25^native(45-94)^**  **(1:0.6 NEC:peptide)** | | |  |  |
| 1 | 1 | 8/100 = 0.08 | 19/100 = 0.19 | 0.11 | 0.11 | 16/100 = 0.16 | 0.08 | 0.06 | 71 | 56 |
|  | 2 | 9/100 = 0.09 | 18/100 = 0.18 | 0.09 |  | 17/100 = 0.17 | 0.08 |  | 71 |  |
|  | 3 | 8/100 = 0.08 | 22/100 = 0.22 | 0.14 |  | 11/100 = 0.11 | 0.03 |  | 26 |  |
| 2 | 1 | 6/100 = 0.06 | 19/100 = 0.19 | 0.13 | 0.14 | 13/95 = 0.14 | 0.08 | 0.08 | 56 | 58 |
|  | 2 | 7/100 = 0.07 | 19/100 = 0.19 | 0.12 |  | 13/100 = 0.13 | 0.06 |  | 44 |  |
|  | 3 | 2/100 = 0.02 | 18/100 = 0.18 | 0.16 |  | 12/100 = 0.12 | 0.10 |  | 73 |  |
| 3 | 1 | 1/100 = 0.01 | 17/90 = 0.19 | 0.18 | 0.14 | 10/100 = 0.10 | 0.09 | 0.07 | 63 | 49 |
|  | 2 | 6/100 = 0.06 | 18/100 = 0.18 | 0.14 |  | 11/100 = 0.11 | 0.05 |  | 35 |  |
|  | 3 | 5/100 = 0.05 | 18/100 = 0.18 | 0.13 |  | 12/100 = 0.12 | 0.07 |  | 49 |  |
|  |  |  | **NEC220** | | | **NEC220 + UL25^native(45-94)^**  **(1:1 NEC:peptide)** | | |  |  |
| 1 | 1 | 8/100 = 0.08 | 19/100 = 0.19 | 0.11 | 0.11 | 10/100 = 0.10 | 0.02 | 0.04 | 18 | 36 |
|  | 2 | 9/100 = 0.09 | 18/100 = 0.18 | 0.09 |  | 13/97 = 0.13 | 0.04 |  | 39 |  |
|  | 3 | 8/100 = 0.08 | 22/100 = 0.22 | 0.14 |  | 14/100 = 0.14 | 0.06 |  | 53 |  |
| 2 | 1 | 3/90 = 0.03 | 10/81 = 0.12 | 0.09 | 0.09 | 2/80 = 0.03 | 0.00 | 0.02 | 9 | 19 |
|  | 2 | 3/100 = 0.03 | 12/90 = 0.13 | 0.10 |  | 2/75 = 0.03 | 0.00 |  | 3 |  |
|  | 3 | 3/85 = 0.04 | 9/80 = 0.11 | 0.07 |  | 7/80 = 0.09 | 0.07 |  | 58 |  |
| 3 | 1 | 5/100 = 0.05 | 12/100 = 0.12 | 0.07 | 0.10 | 7/100 = 0.07 | 0.02 | 0.03 | 21 | 27 |
|  | 2 | 6/100 = 0.06 | 16/105 = 0.15 | 0.09 |  | 8/100 = 0.08 | 0.02 |  | 21 |  |
|  | 3 | 5/100 = 0.05 | 18/100 = 0.18 | 0.13 |  | 9/100 = 0.09 | 0.04 |  | 41 |  |
|  |  |  | **NEC220** | | | **NEC220 + UL25^native(45-94)^**  **(1:10 NEC:peptide)** | | |  |  |
| 1 | 1 | 3/96 = 0.03 | 23/99 = 0.23 | 0.20 | 0.15 | 11/100 = 0.11 | 0.08 | 0.05 | 52 | 30 |
|  | 2 | 7/95 = 0.07 | 22/100 = 0.22 | 0.15 |  | 7/100 = 0.07 | 0.00 |  | 0 |  |
|  | 3 | 9/100 = 0.09 | 20/100 = 0.20 | 0.11 |  | 15/100 = 0.15 | 0.06 |  | 39 |  |
| 2 | 1 | 3/100 = 0.03 | 13/94 = 0.14 | 0.11 | 0.07 | 3/100 = 0.03 | 0.00 | 0.02 | 0 | 33 |
|  | 2 | 4/100 = 0.04 | 8/90 = 0.09 | 0.05 |  | 5/91 = 0.05 | 0.01 |  | 23 |  |
|  | 3 | 5/100 = 0.05 | 8/91 = 0.09 | 0.04 |  | 7/70 = 0.10 | 0.05 |  | 77 |  |
| 3 | 1 | 5/100 = 0.05 | 12/100 = 0.12 | 0.07 | 0.10 | 8/100 = 0.08 | 0.03 | 0.03 | 31 | 34 |
|  | 2 | 6/100 = 0.06 | 16/105 = 0.15 | 0.09 |  | 9/100 = 0.09 | 0.03 |  | 31 |  |
|  | 3 | 5/100 = 0.05 | 18/100 = 0.18 | 0.13 |  | 9/100 = 0.09 | 0.04 |  | 41 |  |
|  |  |  | **NEC220** | | | **NEC220 + UL25^scr(45-94)^**  **(1:0.1 NEC:peptide)** | | |  |  |
| 1 | 1 | 10/100 = 0.10 | 17/100 = 0.17 | 0.07 | 0.09 | 21/100 = 0.21 | 0.11 | 0.10 | 127 | 115 |
|  | 2 | 6/100 = 0.06 | 15/100 = 0.15 | 0.09 |  | 13/100 = 0.13 | 0.07 |  | 81 |  |
|  | 3 | 8/100 = 0.08 | 18/100 = 0.18 | 0.10 |  | 20/100 = 0.20 | 0.12 |  | 138 |  |
| 2 | 1 | 3/96 = 0.03 | 23/99 = 0.23 | 0.20 | 0.15 | 17/100 = 0.17 | 0.14 | 0.13 | 91 | 84 |
|  | 2 | 7/95 = 0.07 | 22/100 = 0.22 | 0.15 |  | 18/100 = 0.18 | 0.11 |  | 70 |  |
|  | 3 | 9/100 = 0.09 | 20/100 = 0.20 | 0.11 |  | 23/100 =0.23 | 0.14 |  | 92 |  |
| 3 | 1 | 6/100 = 0.06 | 19/100 = 0.19 | 0.13 | 0.14 | 19/96 = 0.20 | 0.14 | 0.14 | 101 | 104 |
|  | 2 | 7/100 = 0.07 | 19/100 = 0.19 | 0.12 |  | 18/100 = 0.18 | 0.11 |  | 80 |  |
|  | 3 | 2/100 = 0.02 | 18/100 = 0.18 | 0.16 |  | 20/100 = 0.20 | 0.18 |  | 132 |  |
|  |  |  | **NEC220** | | | **NEC220 + UL25^scr(45-94)^**  **(1:0.3 NEC:peptide)** | | |  |  |
| 1 | 1 | 3/96 = 0.03 | 23/99 = 0.23 | 0.20 | 0.15 | 20/100 = 0.20 | 0.17 | 0.10 | 111 | 65 |
|  | 2 | 7/95 = 0.07 | 22/100 = 0.22 | 0.15 |  | 13/100 = 0.13 | 0.08 |  | 37 |  |
|  | 3 | 9/100 = 0.09 | 20/100 = 0.20 | 0.11 |  | 16/98 = 0.16 | 0.07 |  | 48 |  |
| 2 | 1 | 6/100 = 0.06 | 19/100 = 0.19 | 0.13 | 0.14 | 14/90 = 0.16 | 0.10 | 0.11 | 70 | 82 |
|  | 2 | 7/100 = 0.07 | 19/100 = 0.19 | 0.12 |  | 18/100 = 0.18 | 0.11 |  | 80 |  |
|  | 3 | 2/100 = 0.02 | 18/100 = 0.18 | 0.16 |  | 15/100 = 0.15 | 0.13 |  | 95 |  |
| 3 | 1 | 1/100 = 0.01 | 17/90 = 0.19 | 0.18 | 0.14 | 15/100 = 0.15 | 0.14 | 0.11 | 98 | 77 |
|  | 2 | 6/100 = 0.06 | 18/100 = 0.18 | 0.14 |  | 15/100 = 0.15 | 0.09 |  | 63 |  |
|  | 3 | 5/100 = 0.05 | 18/100 = 0.18 | 0.13 |  | 15/100 = 0.15 | 0.10 |  | 70 |  |
|  |  |  | **NEC220** | | | **NEC220 + UL25^scr(45-94)^**  **(1:0.6 NEC:peptide)** | | |  |  |
| 1 | 1 | 8/100 = 0.08 | 19/100 = 0.19 | 0.11 | 0.11 | 15/90 = 0.17 |  | 0.07 | 76 | 61 |
|  | 2 | 9/100 = 0.09 | 18/100 = 0.18 | 0.09 |  | 15/100 = 0.15 | 0.06 |  | 52 |  |
|  | 3 | 8/100 = 0.08 | 22/100 = 0.22 | 0.14 |  | 14/100 = 0.14 | 0.06 |  | 53 |  |
| 2 | 1 | 6/100 = 0.06 | 19/100 = 0.19 | 0.13 | 0.14 | 13/100 = 0.13 | 0.07 | 0.08 | 51 | 62 |
|  | 2 | 7/100 = 0.07 | 19/100 = 0.19 | 0.12 |  | 12/90 = 0.13 | 0.06 |  | 46 |  |
|  | 3 | 2/100 = 0.02 | 18/100 = 0.18 | 0.16 |  | 14/100 = 0.14 | 0.12 |  | 88 |  |
| 3 | 1 | 1/100 = 0.01 | 17/90 = 0.19 | 0.18 | 0.14 | 12/100 = 0.12 | 0.11 | 0.08 | 78 | 56 |
|  | 2 | 6/100 = 0.06 | 18/100 = 0.18 | 0.14 |  | 11/100 = 0.11 | 0.05 |  | 35 |  |
|  | 3 | 5/100 = 0.05 | 18/100 = 0.18 | 0.13 |  | 13/100 = 0.13 | 0.08 |  | 56 |  |
|  |  |  | **NEC220** | | | **NEC220 + UL25^scr(45-94)^**  **(1:1 NEC:peptide)** | | |  |  |
| 1 | 1 | 8/100 = 0.08 | 19/100 = 0.19 | 0.11 | 0.11 | 13/100 = 0.13 | 0.05 | 0.04 | 44 | 38 |
|  | 2 | 9/100 = 0.09 | 18/100 = 0.18 | 0.09 |  | 13/100 = 0.13 | 0.04 |  | 35 |  |
|  | 3 | 8/100 = 0.08 | 22/100 = 0.22 | 0.14 |  | 12/100 = 0.12 | 0.04 |  | 35 |  |
| 2 | 1 | 3/90 = 0.03 | 10/81 = 0.12 | 0.09 | 0.09 | 7/76 = 0.09 | 0.06 | 0.02 | 65 | 27 |
|  | 2 | 3/100 = 0.03 | 12/90 = 0.13 | 0.10 |  | 4/100 = 0.04 | 0.01 |  | 11 |  |
|  | 3 | 3/85 = 0.04 | 9/80 = 0.11 | 0.07 |  | 4/100 = 0.04 | 0.00 |  | 5 |  |
| 3 | 1 | 5/100 = 0.05 | 12/100 = 0.12 | 0.07 | 0.10 | 11/100 = 0.11 | 0.06 | 0.02 | 62 | 25 |
|  | 2 | 6/100 = 0.06 | 16/105 = 0.15 | 0.09 |  | 6/97 = 0.06 | 0.00 |  | 2 |  |
|  | 3 | 5/100 = 0.05 | 18/100 = 0.18 | 0.13 |  | 6/100 = 0.06 | 0.01 |  | 10 |  |
|  |  |  | **NEC220** | | | **NEC220 + UL25^scr(45-94)^**  **(1:10 NEC:peptide)** | | |  |  |
| 1 | 1 | 3/96 = 0.03 | 23/99 = 0.23 | 0.20 | 0.15 | 9/95 = 0.09 | 0.06 | 0.05 | 42 | 31 |
|  | 2 | 7/95 = 0.07 | 22/100 = 0.22 | 0.15 |  | 13/100 = 0.13 | 0.08 |  | 37 |  |
|  | 3 | 9/100 = 0.09 | 20/100 = 0.20 | 0.11 |  | 11/100 = 0.11 | 0.02 |  | 13 |  |
| 2 | 1 | 3/100 = 0.03 | 13/94 = 0.14 | 0.11 | 0.07 | 6/100 = 0.06 | 0.03 | 0.02 | 46 | 29 |
|  | 2 | 4/100 = 0.04 | 8/90 = 0.09 | 0.05 |  | 5/81 = 0.06 | 0.02 |  | 33 |  |
|  | 3 | 5/100 = 0.05 | 8/91 = 0.09 | 0.04 |  | 4/72 = 0.06 | 0.01 |  | 9 |  |
| 3 | 1 | 5/100 = 0.05 | 12/100 = 0.12 | 0.07 | 0.10 | 9/100 = 0.09 | 0.04 | 0.03 | 41 | 32 |
|  | 2 | 6/100 = 0.06 | 16/105 = 0.15 | 0.09 |  | 7/100 = 0.07 | 0.01 |  | 10 |  |
|  | 3 | 5/100 = 0.05 | 18/100 = 0.18 | 0.13 |  | 9/95 = 0.09 | 0.04 |  | 46 |  |
|  |  |  | **NEC220** | | | **NEC220 + UL25^74-94^**  **(1:0.1 NEC:peptide)** | | |  |  |
| 1 | 1 | 10/100 = 0.10 | 17/100 = 0.17 | 0.07 | 0.09 | 15/100 = 0.15 | 0.05 | 0.08 | 58 | 96 |
|  | 2 | 6/100 = 0.06 | 15/100 = 0.15 | 0.09 |  | 17/100 = 0.17 | 0.11 |  | 127 |  |
|  | 3 | 8/100 = 0.08 | 18/100 = 0.18 | 0.10 |  | 17/100 = 0.17 | 0.09 |  | 104 |  |
| 2 | 1 | 3/96 = 0.03 | 23/99 = 0.23 | 0.20 | 0.15 | 21/100 = 0.21 | 0.18 | 0.15 | 117 | 97 |
|  | 2 | 7/95 = 0.07 | 22/100 = 0.22 | 0.15 |  | 23/100 = 0.23 | 0.16 |  | 102 |  |
|  | 3 | 9/100 = 0.09 | 20/100 = 0.20 | 0.11 |  | 20/100 = 0.20 | 0.11 |  | 72 |  |
| 3 | 1 | 6/100 = 0.06 | 19/100 = 0.19 | 0.13 | 0.14 | 17/90 = 0.19 | 0.13 | 0.13 | 94 | 97 |
|  | 2 | 7/100 = 0.07 | 19/100 = 0.19 | 0.12 |  | 21/100 = 0.21 | 0.14 |  | 102 |  |
|  | 3 | 2/100 = 0.02 | 18/100 = 0.18 | 0.16 |  | 15/100 = 0.15 | 0.13 |  | 95 |  |
|  |  |  | **NEC220** | | | **NEC220 + UL25^74-94^**  **(1:0.3 NEC:peptide)** | | |  |  |
| 1 | 1 | 3/96 = 0.03 | 23/99 = 0.23 | 0.20 | 0.15 | 25/100 = 0.25 | 0.22 | 0.16 | 143 | 102 |
|  | 2 | 7/95 = 0.07 | 22/100 = 0.22 | 0.15 |  | 23/100 = 0.23 | 0.16 |  | 103 |  |
|  | 3 | 9/100 = 0.09 | 20/100 = 0.20 | 0.11 |  | 18/100 = 0.18 | 0.09 |  | 59 |  |
| 2 | 1 | 6/100 = 0.06 | 19/100 = 0.19 | 0.13 | 0.14 | 17/100 = 0.17 | 0.11 | 0.13 | 80 | 98 |
|  | 2 | 7/100 = 0.07 | 19/100 = 0.19 | 0.12 |  | 17/100 = 0.17 | 0.11 |  | 73 |  |
|  | 3 | 2/100 = 0.02 | 18/100 = 0.18 | 0.16 |  | 21/100 = 0.21 | 0.19 |  | 139 |  |
| 3 | 1 | 1/100 = 0.01 | 17/90 = 0.19 | 0.18 | 0.14 | 17/90 = 0.19 | 0.18 | 0.14 | 125 | 98 |
|  | 2 | 6/100 = 0.06 | 18/100 = 0.18 | 0.14 |  | 17/100 = 0.17 | 0.11 |  | 77 |  |
|  | 3 | 5/100 = 0.05 | 18/100 = 0.18 | 0.13 |  | 18/100 = 0.18 | 0.13 |  | 91 |  |
|  |  |  | **NEC220** | | | **NEC220 + UL25^74-94^**  **(1:0.6 NEC:peptide)** | | |  |  |
| 1 | 1 | 6/100 = 0.06 | 19/100 = 0.19 | 0.13 | 0.14 | 18/100 = 0.18 | 0.12 | 0.12 | 88 | 87 |
|  | 2 | 7/100 = 0.07 | 19/100 = 0.19 | 0.12 |  | 16/95 = 0.17 | 0.10 |  | 72 |  |
|  | 3 | 2/100 = 0.02 | 18/100 = 0.18 | 0.16 |  | 16/100 = 0.16 | 0.14 |  | 102 |  |
| 2 | 1 | 1/100 = 0.01 | 17/90 = 0.19 | 0.18 | 0.14 | 18/93 = 0.19 | 0.18 | 0.15 | 128 | 103 |
|  | 2 | 6/100 = 0.06 | 18/100 = 0.18 | 0.14 |  | 20/100 = 0.20 | 0.14 |  | 98 |  |
|  | 3 | 5/100 = 0.05 | 18/100 = 0.18 | 0.13 |  | 17/100 = 0.17 | 0.12 |  | 84 |  |
| 3 | 1 | 8/100 = 0.08 | 19/100 = 0.19 | 0.11 | 0.11 | 17/100 = 0.17 | 0.09 | 0.10 | 80 | 90 |
|  | 2 | 9/100 = 0.09 | 18/100 = 0.18 | 0.09 |  | 20/100 = 0.20 | 0.11 |  | 97 |  |
|  | 3 | 8/100 = 0.08 | 22/100 = 0.22 | 0.14 |  | 18/96 = 0.19 | 0.11 |  | 95 |  |
|  |  |  | **NEC220** | | | **NEC220 + UL25^74-94^**  **(1:1 NEC:peptide)** | | |  |  |
| 1 | 1 | 8/100 = 0.08 | 19/100 = 0.19 | 0.11 | 0.11 | 15/96 = 0.16 | 0.08 | 0.09 | 67 | 75 |
|  | 2 | 9/100 = 0.09 | 18/100 = 0.18 | 0.09 |  | 18/100 = 0.18 | 0.09 |  | 79 |  |
|  | 3 | 8/100 = 0.08 | 22/100 = 0.22 | 0.14 |  | 17/100 = 0.17 | 0.09 |  | 79 |  |
| 2 | 1 | 1/100 = 0.01 | 17/90 = 0.19 | 0.18 | 0.14 | 16/100 = 0.16 | 0.15 | 0.13 | 105 | 93 |
|  | 2 | 6/100 = 0.06 | 18/100 = 0.18 | 0.14 |  | 18/100 = 0.18 | 0.12 |  | 84 |  |
|  | 3 | 5/100 = 0.05 | 18/100 = 0.18 | 0.13 |  | 18/100 = 0.18 | 0.13 |  | 91 |  |
| 3 | 1 | 5/100 = 0.05 | 12/100 = 0.12 | 0.07 | 0.10 | 10/100 = 0.10 | 0.05 | 0.05 | 51 | 55 |
|  | 2 | 6/100 = 0.06 | 16/105 = 0.15 | 0.09 |  | 10/100 = 0.10 | 0.04 |  | 41 |  |
|  | 3 | 5/100 = 0.05 | 18/100 = 0.18 | 0.13 |  | 12/100 = 0.12 | 0.07 |  | 72 |  |
|  |  |  | **NEC220** | | | **NEC220 + UL25^74-94^**  **(1:10 NEC:peptide)** | | |  |  |
| 1 | 1 | 3/96 = 0.03 | 23/99 = 0.23 | 0.20 | 0.15 | 17/100 = 0.17 | 0.14 | 0.10 | 91 | 64 |
|  | 2 | 7/95 = 0.07 | 22/100 = 0.22 | 0.15 |  | 14/96 = 0.15 | 0.08 |  | 47 |  |
|  | 3 | 9/100 = 0.09 | 20/100 = 0.20 | 0.11 |  | 17/100 = 0.17 | 0.08 |  | 52 |  |
| 2 | 1 | 3/100 = 0.03 | 13/94 = 0.14 | 0.11 | 0.07 | 6/97 = 0.06 | 0.03 | 0.04 | 49 | 66 |
|  | 2 | 4/100 = 0.04 | 8/90 = 0.09 | 0.05 |  | 6/100 = 0.06 | 0.02 |  | 31 |  |
|  | 3 | 5/100 = 0.05 | 8/91 = 0.09 | 0.04 |  | 12/95 = 0.13 | 0.08 |  | 117 |  |
| 3 | 1 | 5/100 = 0.05 | 12/100 = 0.12 | 0.07 | 0.10 | 15/100 = 0.15 | 0.10 | 0.07 | 103 | 72 |
|  | 2 | 6/100 = 0.06 | 16/105 = 0.15 | 0.09 |  | 11/100 = 0.11 | 0.05 |  | 51 |  |
|  | 3 | 5/100 = 0.05 | 18/100 = 0.18 | 0.13 |  | 11/100 = 0.11 | 0.06 |  | 62 |  |
|  |  |  | **NEC220** | | | **NEC220 + gB RC^103-152^**  **(1:0.1 NEC:peptide)** | | |  |  |
| 1 | 1 | 10/100 = 0.10 | 17/100 = 0.17 | 0.07 | 0.09 | 20/100 = 0.20 | 0.10 | 0.10 | 115 | 115 |
|  | 2 | 6/100 = 0.06 | 15/100 = 0.15 | 0.09 |  | 17/100 = 0.17 | 0.11 |  | 127 |  |
|  | 3 | 8/100 = 0.08 | 18/100 = 0.18 | 0.10 |  | 17/100 = 0.17 | 0.09 |  | 104 |  |
| 2 | 1 | 3/96 = 0.03 | 23/99 = 0.23 | 0.20 | 0.15 | 22/100 = 0.22 | 0.19 | 0.18 | 124 | 120 |
|  | 2 | 7/95 = 0.07 | 22/100 = 0.22 | 0.15 |  | 25/98 = 0.26 | 0.19 |  | 119 |  |
|  | 3 | 9/100 = 0.09 | 20/100 = 0.20 | 0.11 |  | 24/90 = 0.27 | 0.18 |  | 116 |  |
| 3 | 1 | 6/100 = 0.06 | 19/100 = 0.19 | 0.13 | 0.14 | 19/90 = 0.21 | 0.15 | 0.15 | 111 | 107 |
|  | 2 | 7/100 = 0.07 | 19/100 = 0.19 | 0.12 |  | 16/100 = 0.16 | 0.09 |  | 66 |  |
|  | 3 | 2/100 = 0.02 | 18/100 = 0.18 | 0.16 |  | 21/97 = 0.22 | 0.20 |  | 144 |  |
|  |  |  | **NEC220** | | | **NEC220 + gB RC^103-152^**  **(1:0.3 NEC:peptide)** | | |  |  |
| 1 | 1 | 3/96 = 0.03 | 23/99 = 0.23 | 0.20 | 0.15 | 21/100 = 0.21 | 0.18 | 0.16 | 117 | 102 |
|  | 2 | 7/95 = 0.07 | 22/100 = 0.22 | 0.15 |  | 23/100 = 0.23 | 0.16 |  | 103 |  |
|  | 3 | 9/100 = 0.09 | 20/100 = 0.20 | 0.11 |  | 22/100 = 0.22 | 0.13 |  | 85 |  |
| 2 | 1 | 6/100 = 0.06 | 19/100 = 0.19 | 0.13 | 0.14 | 18/100 = 0.18 | 0.12 | 0.14 | 88 | 105 |
|  | 2 | 7/100 = 0.07 | 19/100 = 0.19 | 0.12 |  | 21/100 = 0.21 | 0.14 |  | 102 |  |
|  | 3 | 2/100 = 0.02 | 18/100 = 0.18 | 0.16 |  | 19/100 = 0.19 | 0.17 |  | 124 |  |
| 3 | 1 | 1/100 = 0.01 | 17/90 = 0.19 | 0.18 | 0.14 | 17/100 = 0.17 | 0.16 | 0.14 | 112 | 96 |
|  | 2 | 6/100 = 0.06 | 18/100 = 0.18 | 0.14 |  | 18/100 = 0.18 | 0.12 |  | 84 |  |
|  | 3 | 5/100 = 0.05 | 18/100 = 0.18 | 0.13 |  | 17/100 = 0.17 | 0.12 |  | 91 |  |
|  |  |  | **NEC220** | | | **NEC220 + gB RC^103-152^**  **(1:0.6 NEC:peptide)** | | |  |  |
| 1 | 1 | 6/100 = 0.06 | 19/100 = 0.19 | 0.13 | 0.14 | 16/100 = 0.16 | 0.10 | 0.12 | 73 | 85 |
|  | 2 | 7/100 = 0.07 | 19/100 = 0.19 | 0.12 |  | 14/100 = 0.14 | 0.07 |  | 51 |  |
|  | 3 | 2/100 = 0.02 | 18/100 = 0.18 | 0.16 |  | 20/100 = 0.20 | 0.18 |  | 132 |  |
| 2 | 1 | 1/100 = 0.01 | 17/90 = 0.19 | 0.18 | 0.14 | 16/100 = 0.16 | 0.15 | 0.13 | 105 | 93 |
|  | 2 | 6/100 = 0.06 | 18/100 = 0.18 | 0.14 |  | 19/100 = 0.19 | 0.13 |  | 91 |  |
|  | 3 | 5/100 = 0.05 | 18/100 = 0.18 | 0.13 |  | 16/94 = 0.17 | 0.12 |  | 84 |  |
| 3 | 1 | 8/100 = 0.08 | 19/100 = 0.19 | 0.11 | 0.11 | 16/100 = 0.16 | 0.08 | 0.10 | 71 | 91 |
|  | 2 | 9/100 = 0.09 | 18/100 = 0.18 | 0.09 |  | 18/100 = 0.18 | 0.09 |  | 80 |  |
|  | 3 | 8/100 = 0.08 | 22/100 = 0.22 | 0.14 |  | 22/100 = 0.22 | 0.14 |  | 124 |  |
|  |  |  | **NEC220** | | | **NEC220 + gB RC^103-152^**  **(1:1 NEC:peptide)** | | |  |  |
| 1 | 1 | 8/100 = 0.08 | 19/100 = 0.19 | 0.11 | 0.11 | 18/95 = 0.19 | 0.11 | 0.12 | 97 | 103 |
|  | 2 | 9/100 = 0.09 | 18/100 = 0.18 | 0.09 |  | 19/100 = 0.19 | 0.10 |  | 88 |  |
|  | 3 | 8/100 = 0.08 | 22/100 = 0.22 | 0.14 |  | 22/100 = 0.22 | 0.14 |  | 124 |  |
| 2 | 1 | 1/100 = 0.01 | 17/90 = 0.19 | 0.18 | 0.14 | 17/100 = 0.17 | 0.16 | 0.14 | 112 | 98 |
|  | 2 | 6/100 = 0.06 | 18/100 = 0.18 | 0.14 |  | 16/100 = 0.16 | 0.10 |  | 70 |  |
|  | 3 | 5/100 = 0.05 | 18/100 = 0.18 | 0.13 |  | 19/90 = 0.21 | 0.16 |  | 113 |  |
| 3 | 1 | 5/100 = 0.05 | 12/100 = 0.12 | 0.07 | 0.10 | 13/100 = 0.13 | 0.08 | 0.08 | 82 | 82 |
|  | 2 | 6/100 = 0.06 | 16/105 = 0.15 | 0.09 |  | 15/100 = 0.15 | 0.09 |  | 92 |  |
|  | 3 | 5/100 = 0.05 | 18/100 = 0.18 | 0.13 |  | 12/100 = 0.12 | 0.07 |  | 72 |  |
|  |  |  | **NEC220** | | | **NEC220 + gB RC^103-152^**  **(1:10 NEC:peptide)** | | |  |  |
| 1 | 1 | 3/96 = 0.03 | 23/99 = 0.23 | 0.20 | 0.15 | 20/91 = 0.22 | 0.19 | 0.12 | 124 | 82 |
|  | 2 | 7/95 = 0.07 | 22/100 = 0.22 | 0.15 |  | 19/100 = 0.19 | 0.12 |  | 76 |  |
|  | 3 | 9/100 = 0.09 | 20/100 = 0.20 | 0.11 |  | 16/100 = 0.16 | 0.07 |  | 46 |  |
| 2 | 1 | 3/100 = 0.03 | 13/94 = 0.14 | 0.11 | 0.07 | 9/90 = 0.10 | 0.07 | 0.05 | 108 | 73 |
|  | 2 | 4/100 = 0.04 | 8/90 = 0.09 | 0.05 |  | 8/90 = 0.09 | 0.05 |  | 75 |  |
|  | 3 | 5/100 = 0.05 | 8/91 = 0.09 | 0.04 |  | 5/67 = 0.07 | 0.02 |  | 38 |  |
| 3 | 1 | 5/100 = 0.05 | 12/100 = 0.12 | 0.07 | 0.10 | 9/97 = 0.09 | 0.04 | 0.06 | 44 | 63 |
|  | 2 | 6/100 = 0.06 | 16/105 = 0.15 | 0.09 |  | 12/100 = 0.12 | 0.06 |  | 62 |  |
|  | 3 | 5/100 = 0.05 | 18/100 = 0.18 | 0.13 |  | 13/100 = 0.13 | 0.08 |  | 82 |  |
|  |  |  | **NEC220** | | | **NEC220 + (EAEKAAK)_7_EAEK**  **(1:0.1 NEC:peptide)** | | |  |  |
| 1 | 1 | 3/96 = 0.03 | 23/99 = 0.23 | 0.20 | 0.15 | 22/100 = 0.22 | 0.19 | 0.15 | 124 | 97 |
|  | 2 | 7/95 = 0.07 | 22/100 = 0.22 | 0.15 |  | 24/100 = 0.24 | 0.17 |  | 109 |  |
|  | 3 | 9/100 = 0.09 | 20/100 = 0.20 | 0.11 |  | 18/100 = 0.18 | 0.09 |  | 59 |  |
| 2 | 1 | 6/100 = 0.06 | 19/100 = 0.19 | 0.13 | 0.14 | 21/93 = 0.23 | 0.17 | 0.15 | 121 | 109 |
|  | 2 | 7/100 = 0.07 | 19/100 = 0.19 | 0.12 |  | 19/100 = 0.19 | 0.12 |  | 88 |  |
|  | 3 | 2/100 = 0.02 | 18/100 = 0.18 | 0.16 |  | 18/100 = 0.18 | 0.16 |  | 117 |  |
| 3 | 1 | 8/100 = 0.08 | 19/100 = 0.19 | 0.11 | 0.11 | 17/100 = 0.17 | 0.09 | 0.09 | 80 | 82 |
|  | 2 | 9/100 = 0.09 | 18/100 = 0.18 | 0.09 |  | 16/100 = 0.16 | 0.07 |  | 62 |  |
|  | 3 | 8/100 = 0.08 | 22/100 = 0.22 | 0.14 |  | 20/100 = 0.20 | 0.12 |  | 106 |  |
|  |  |  | **NEC220** | | | **NEC220 + (EAEKAAK)_7_EAEK**  **(1:0.3 NEC:peptide)** | | |  |  |
| 1 | 1 | 3/96 = 0.03 | 23/99 = 0.23 | 0.20 | 0.15 | 23/100 = 0.23 | 0.20 | 0.15 | 130 | 99 |
|  | 2 | 7/95 = 0.07 | 22/100 = 0.22 | 0.15 |  | 20/100 = 0.20 | 0.13 |  | 83 |  |
|  | 3 | 9/100 = 0.09 | 20/100 = 0.20 | 0.11 |  | 21/96 = 0.22 | 0.13 |  | 84 |  |
| 2 | 1 | 6/100 = 0.06 | 19/100 = 0.19 | 0.13 | 0.14 | 20/100 = 0.20 | 0.14 | 0.13 | 102 | 95 |
|  | 2 | 7/100 = 0.07 | 19/100 = 0.19 | 0.12 |  | 18/100 = 0.18 | 0.11 |  | 80 |  |
|  | 3 | 2/100 = 0.02 | 18/100 = 0.18 | 0.16 |  | 16/100 = 0.16 | 0.14 |  | 102 |  |
| 3 | 1 | 1/100 = 0.01 | 17/90 = 0.19 | 0.18 | 0.14 | 17/100 = 0.17 | 0.16 | 0.14 | 112 | 96 |
|  | 2 | 6/100 = 0.06 | 18/100 = 0.18 | 0.14 |  | 16/100 = 0.16 | 0.10 |  | 70 |  |
|  | 3 | 5/100 = 0.05 | 18/100 = 0.18 | 0.13 |  | 20/100 = 0.20 | 0.15 |  | 105 |  |
|  |  |  | **NEC220** | | | **NEC220 + (EAEKAAK)_7_EAEK**  **(1:0.6 NEC:peptide)** | | |  |  |
| 1 | 1 | 6/100 = 0.06 | 19/100 = 0.19 | 0.13 | 0.14 | 18/100 = 0.18 | 0.12 | 0.12 | 88 | 88 |
|  | 2 | 7/100 = 0.07 | 19/100 = 0.19 | 0.12 |  | 15/100 = 0.15 | 0.08 |  | 58 |  |
|  | 3 | 2/100 = 0.02 | 18/100 = 0.18 | 0.16 |  | 18/100 = 0.18 | 0.16 |  | 117 |  |
| 2 | 1 | 1/100 = 0.01 | 17/90 = 0.19 | 0.18 | 0.14 | 19/100 = 0.19 | 0.18 | 0.16 | 126 | 112 |
|  | 2 | 6/100 = 0.06 | 18/100 = 0.18 | 0.14 |  | 21/100 = 0.21 | 0.15 |  | 105 |  |
|  | 3 | 5/100 = 0.05 | 18/100 = 0.18 | 0.13 |  | 20/100 = 0.20 | 0.15 |  | 105 |  |
| 3 | 1 | 8/100 = 0.08 | 19/100 = 0.19 | 0.11 | 0.11 | 16/95 = 0.17 | 0.09 | 0.10 | 78 | 85 |
|  | 2 | 9/100 = 0.09 | 18/100 = 0.18 | 0.09 |  | 15/100 = 0.15 | 0.06 |  | 53 |  |
|  | 3 | 8/100 = 0.08 | 22/100 = 0.22 | 0.14 |  | 22/100 = 0.22 | 0.14 |  | 124 |  |
|  |  |  | **NEC220** | | | **NEC220 + (EAEKAAK)_7_EAEK**  **(1:1 NEC:peptide)** | | |  |  |
| 1 | 1 | 8/100 = 0.08 | 19/100 = 0.19 | 0.11 | 0.11 | 19/90 = 0.21 | 0.13 | 0.12 | 116 | 106 |
|  | 2 | 9/100 = 0.09 | 18/100 = 0.18 | 0.09 |  | 21/100 = 0.21 | 0.12 |  | 106 |  |
|  | 3 | 8/100 = 0.08 | 22/100 = 0.22 | 0.14 |  | 19/100 = 0.19 | 0.08 |  | 97 |  |
| 2 | 1 | 6/100 = 0.06 | 19/100 = 0.19 | 0.13 | 0.14 | 18/100 = 0.18 | 0.12 | 0.12 | 88 | 90 |
|  | 2 | 7/100 = 0.07 | 19/100 = 0.19 | 0.12 |  | 17/100 = 0.17 | 0.10 |  | 73 |  |
|  | 3 | 2/100 = 0.02 | 18/100 = 0.18 | 0.16 |  | 17/100 = 0.17 | 0.15 |  | 110 |  |
| 3 | 1 | 1/100 = 0.01 | 17/90 = 0.19 | 0.18 | 0.14 | 15/90 = 0.17 | 0.16 | 0.16 | 110 | 102 |
|  | 2 | 6/100 = 0.06 | 18/100 = 0.18 | 0.14 |  | 18/100 = 0.18 | 0.12 |  | 84 |  |
|  | 3 | 5/100 = 0.05 | 18/100 = 0.18 | 0.13 |  | 21/100 = 0.21 | 0.16 |  | 112 |  |
|  |  |  | **NEC220** | | | **NEC220 + (EAEKAAK)_7_EAEK**  **(1:10 NEC:peptide)** | | |  |  |
| 1 | 1 | 9/100 = 0.09 | 0.04 | 0.05 | 0.06 | 19/99 = 0.19 | 0.15 | 0.15 | 34 | 40 |
|  | 2 | 11/100 = 0.11 | 0.04 | 0.07 |  | 16/100 = 0.16 | 0.12 |  | 45 |  |
|  | 3 | 10/100 = 0.10 | 0.04 | 0.06 |  | 21/100 = 0.21 | 0.17 |  | 41 |  |
| 2 | 1 | 15/101 = 0.15 | 0.06 | 0.09 | 0.11 | 26/100 = 0.26 | 0.20 | 0.21 | 41 | 53 |
|  | 2 | 19/98 = 0.19 | 0.06 | 0.13 |  | 22/100 = 0.22 | 0.16 |  | 63 |  |
|  | 3 | 14/100 = 0.14 | 0.02 | 0.12 |  | 30/100 = 0.30 | 0.28 |  | 56 |  |
| 3 | 1 | 9/100 = 0.09 | 0.04 | 0.05 | 0.08 | 17/97 = 0.18 | 0.14 | 0.14 | 35 | 54 |
|  | 2 | 12/100 = 0.12 | 0.04 | 0.08 |  | 16/96 = 0.16 | 0.12 |  | 57 |  |
|  | 3 | 13/100 = 0.13 | 0.03 | 0.10 |  | 19/100 = 0.19 | 0.13 |  | 71 |  |
|  |  |  | **NEC220** | | | **NEC220 + UL25∆44 Q72A**  **(1:0.1 NEC:UL25)** | | |  |  |
| 1 | 1 | 10/100 = 0.10 | 17/100 = 0.17 | 0.07 | 0.09 | 14/100 = 0.14 | 0.04 | 0.09 | 46 | 107 |
|  | 2 | 6/100 = 0.06 | 15/100 = 0.15 | 0.09 |  | 17/90 = 0.19 | 0.13 |  | 149 |  |
|  | 3 | 8/100 = 0.08 | 18/100 = 0.18 | 0.10 |  | 19/100 = 0.19 | 0.11 |  | 127 |  |
| 2 | 1 | 3/96 = 0.03 | 23/99 = 0.23 | 0.20 | 0.15 | 21/100 = 0.21 | 0.18 | 0.17 | 117 | 110 |
|  | 2 | 7/95 = 0.07 | 22/100 = 0.22 | 0.15 |  | 28/100 = 0.28 | 0.21 |  | 135 |  |
|  | 3 | 9/100 = 0.09 | 20/100 = 0.20 | 0.11 |  | 21/100 = 0.21 | 0.12 |  | 79 |  |
| 3 | 1 | 6/100 = 0.06 | 19/100 = 0.19 | 0.13 | 0.14 | 17/100 = 0.17 | 0.11 | 0.13 | 80 | 97 |
|  | 2 | 7/100 = 0.07 | 19/100 = 0.19 | 0.12 |  | 14/96 = 0.15 | 0.08 |  | 55 |  |
|  | 3 | 2/100 = 0.02 | 18/100 = 0.18 | 0.16 |  | 23/100 = 0.23 | 0.21 |  | 154 |  |
|  |  |  | **NEC220** | | | **NEC220 + UL25∆44 Q72A**  **(1:0.3 NEC:UL25)** | | |  |  |
| 1 | 1 | 3/96 = 0.03 | 23/99 = 0.23 | 0.20 | 0.15 | 27/97 = 0.28 | 0.25 | 0.15 | 162 | 101 |
|  | 2 | 7/95 = 0.07 | 22/100 = 0.22 | 0.15 |  | 17/100 = 0.17 | 0.10 |  | 63 |  |
|  | 3 | 9/100 = 0.09 | 20/100 = 0.20 | 0.11 |  | 21/100 = 0.21 | 0.11 |  | 79 |  |
| 2 | 1 | 6/100 = 0.06 | 19/100 = 0.19 | 0.13 | 0.14 | 16/100 = 0.16 | 0.10 | 0.12 | 73 | 88 |
|  | 2 | 7/100 = 0.07 | 19/100 = 0.19 | 0.12 |  | 16/100 = 0.16 | 0.09 |  | 66 |  |
|  | 3 | 2/100 = 0.02 | 18/100 = 0.18 | 0.16 |  | 19/100 = 0.19 | 0.17 |  | 124 |  |
| 3 | 1 | 1/100 = 0.01 | 17/90 = 0.19 | 0.18 | 0.14 | 21/100 = 0.21 | 0.20 | 0.14 | 140 | 98 |
|  | 2 | 6/100 = 0.06 | 18/100 = 0.18 | 0.14 |  | 15/100 = 0.15 | 0.09 |  | 63 |  |
|  | 3 | 5/100 = 0.05 | 18/100 = 0.18 | 0.13 |  | 18/100 = 0.18 | 0.13 |  | 91 |  |
|  |  |  | **NEC220** | | | **NEC220 + UL25∆44 Q72A**  **(1:0.6 NEC:UL25)** | | |  |  |
| 1 | 1 | 8/100 = 0.08 | 19/100 = 0.19 | 0.11 | 0.11 | 16/100 = 0.16 | 0.08 | 0.11 | 71 | 97 |
|  | 2 | 9/100 = 0.09 | 18/100 = 0.18 | 0.09 |  | 21/100 = 0.21 | 0.12 |  | 106 |  |
|  | 3 | 8/100 = 0.08 | 22/100 = 0.22 | 0.14 |  | 21/100 = 0.21 | 0.13 |  | 115 |  |
| 2 | 1 | 6/100 = 0.06 | 19/100 = 0.19 | 0.13 | 0.14 | 18/100 = 0.18 | 0.08 | 0.13 | 88 | 93 |
|  | 2 | 7/100 = 0.07 | 19/100 = 0.19 | 0.12 |  | 16/94 = 0.17 | 0.10 |  | 73 |  |
|  | 3 | 2/100 = 0.02 | 18/100 = 0.18 | 0.16 |  | 18/100 = 0.18 | 0.16 |  | 117 |  |
| 3 | 1 | 1/100 = 0.01 | 17/90 = 0.19 | 0.18 | 0.14 | 20/100 = 0.20 | 0.19 | 0.15 | 133 | 105 |
|  | 2 | 6/100 = 0.06 | 18/100 = 0.18 | 0.14 |  | 15/90 = 0.17 | 0.11 |  | 75 |  |
|  | 3 | 5/100 = 0.05 | 18/100 = 0.18 | 0.13 |  | 19/93 = 0.20 | 0.15 |  | 108 |  |
|  |  |  | **NEC220** | | | **NEC220 + UL25∆44 Q72A**  **(1:1 NEC:UL25)** | | |  |  |
| 1 | 1 | 8/100 = 0.08 | 19/100 = 0.19 | 0.11 | 0.11 | 19/91 = 0.21 | 0.13 | 0.13 | 113 | 112 |
|  | 2 | 9/100 = 0.09 | 18/100 = 0.18 | 0.09 |  | 21/80 = 0.27 | 0.18 |  | 152 |  |
|  | 3 | 8/100 = 0.08 | 22/100 = 0.22 | 0.14 |  | 16/100 = 0.16 | 0.08 |  | 71 |  |
| 2 | 1 | 1/100 = 0.01 | 17/90 = 0.19 | 0.18 | 0.14 | 16/100 = 0.16 | 0.15 | 0.15 | 105 | 105 |
|  | 2 | 6/100 = 0.06 | 18/100 = 0.18 | 0.14 |  | 22/100 = 0.22 | 0.16 |  | 112 |  |
|  | 3 | 5/100 = 0.05 | 18/100 = 0.18 | 0.13 |  | 19/100 = 0.19 | 0.14 |  | 98 |  |
| 3 | 1 | 3/90 = 0.03 | 10/81 = 0.12 | 0.09 | 0.09 | 11/97 = 0.11 | 0.08 | 0.09 | 89 | 100 |
|  | 2 | 3/100 = 0.03 | 12/90 = 0.13 | 0.10 |  | 11/90 = 0.12 | 0.09 |  | 102 |  |
|  | 3 | 3/85 = 0.04 | 9/80 = 0.11 | 0.07 |  | 13/97 = 0.13 | 0.09 |  | 109 |  |
|  |  |  | **NEC220** | | | **NEC220 + UL25∆44 Q72A**  **(1:10 NEC:UL25)** | | |  |  |
| 1 | 1 | 3/100 = 0.03 | 13/94 = 0.14 | 0.11 | 0.07 | 4/100 = 0.04 | 0.01 | 0.00 | 15 | 12 |
|  | 2 | 4/100 = 0.04 | 8/90 = 0.09 | 0.05 |  | 4/100 = 0.04 | 0.00 |  | 0 |  |
|  | 3 | 5/100 = 0.05 | 8/91 = 0.09 | 0.04 |  | 5/80 = 0.06 | 0.01 |  | 19 |  |
| 2 | 1 | 5/100 = 0.05 | 12/100 = 0.12 | 0.07 | 0.10 | 5/100 = 0.05 | 0.00 | 0.00 | 0 | 7 |
|  | 2 | 6/100 = 0.06 | 16/105 = 0.15 | 0.09 |  | 6/97 = 0.06 | 0.00 |  | 2 |  |
|  | 3 | 5/100 = 0.05 | 18/100 = 0.18 | 0.13 |  | 7/100 = 0.07 | 0.02 |  | 21 |  |
| 3 | 1 | 3/87 = 0.03 | 17/85 = 0.20 | 0.17 | 0.14 | 3/85 = 0.03 | 0.00 | 0.01 | 0 | 7 |
|  | 2 | 2/96 = 0.02 | 15/94 = 0.16 | 0.14 |  | 5/101 = 0.05 | 0.03 |  | 20 |  |
|  | 3 | 3/103 = 0.03 | 15/97= 0.15 | 0.12 |  | 3/95 = 0.03 | 0.00 |  | 2 |  |

**References**

1. Gautier, R., Douguet, D., Antonny, B. & Drin, G. HELIQUEST: a web server to screen sequences with specific alpha-helical properties. *Bioinformatics (Oxford, England)*. **24**, 2101-2102, doi:10.1093/bioinformatics/btn392 (2008).

2. Whitmore, L. & Wallace, B. A. Protein secondary structure analyses from circular dichroism spectroscopy: methods and reference databases. *Biopolymers*. **89**, 392-400, doi:10.1002/bip.20853 (2008).

3. van Stokkum, I. H., Spoelder, H. J., Bloemendal, M., van Grondelle, R. & Groen, F. C. Estimation of protein secondary structure and error analysis from circular dichroism spectra. *Anal. Biochem.* **191**, 110-118 (1990).

4. Sreerama, N. & Woody, R. W. Estimation of protein secondary structure from circular dichroism spectra: comparison of CONTIN, SELCON, and CDSSTR methods with an expanded reference set. *Anal. Biochem.* **287**, 252-260, doi:10.1006/abio.2000.4880 (2000).
